# The Pathogenesis and Treatment of Rickets

**Published:** 1901-04-20

**Authors:** 


### THE PATHOGENESIS AND TREATMENT OF RICKETS.

It has long been customary to regard rickets as due to a deficiency of some essential constituent in the food. Dr. Eric Pritchard,[1] however, shows cause for believing that it may be due to excess of some necessary element resulting in deficient oxidation and the production of lactic and other organic acids, which are the direct cause by which the symptoms of the disease are produced. He has made a special study of cases occurring among the children of the well-to-do, which, he says, are met with chiefly, although not exclusively, among bottle-fed infants. After describing the symptoms met with in these cases, he justly remarks that it seems impossible to believe that such undoubted cases of rickets in the children of well-to-do parents differ in essence and pathogenesis from those found among the children of the poor. Yet the etiological factors which explain the disease in one case play no obvious part in the other. Any theory of rickets or the causation of rickets ought to cover both sets of cases, and he argues that neither on the hypothesis of the cause being a deficiency of something essential nor of its being the presence of some noxious ingredient in the food, does it hold good for all cases. Turning, however, to the suggestion that the disease is the result of the presence of lactic acid and other acids in the blood, he shows that there is reason to believe that, if present, this would be an efficient cause, and he goes on to show that where an excess of food, especially of carbohydrates, is taken for a considerable time, while at the same time there is deficiency of oxidation, lactic acid is produced in excess. When infants are fed on a diet containing an excess of carbohydrates, so long as they can dispose of the excess by storing it up in the form of glycogen, or by the deposition of fat, they appear to be free from symptoms. The danger zone is, however, reached with the physiological limit of fat deposition, and then (perhaps the seventh or eighth month) serious symptoms supervene. To get rid of the excess of nutriment absorbed the organism must have recourse to oxidation or destructive metabolism Should the oxygen be equal to the emergency metabolism will still convert the carbohydrates into their normal end-products. But should the oxygen supply fail, incomplete metabolites of the nature of lactic acid will be formed instead. Even then, as long as the acid can be neutralised by the alkaline salts in the blood, and the skin, mucous membranes, liver, &c., co-operate in the oxidising or eliminating processes no serious symptoms may result, but should the co-operation of these organs fail from any cause, chill for example, then lactic acid would with difficulty be removed, and the alkalinity of the blood would almost be reduced to zero. Dr. Pritchard traces the manner in which this diminished alkalinity due to the presence of lactic acid may lead to rickets, and concludes that (1) the symptoms of rickets can be explained by the presence of an excess of lactic and other organic and inorganic acids in the blood; (2) excess of lactic acid can be generated when the food supply (carbohydrate chiefly) is relatively excessive, or when the available oxygen is relatively deficient; (3) infants fed on excessive diets can develop rickets though no element necessary for metabolism is absent from the food; (4) such cases can be cured by reducing the food to normal proportions: (5) the cause of rickets in these cases, and probably in all cases, is relative excess of some element, and that element usually carbohydrate. This seems a good workable hypothesis. Once bring ourselves to admit that lactic acid will produce rickets, and all the rest is easy; for evidently many of the conditions which are known to dispose to this disease might equally lead to a development of lactic acid. The real importance of Dr. Pritchard's suggestion lies in its application. According to his teaching the common practice of adding fatty and proteid elements to the diet is not enough; we must also seek for and subtract the elements which are being given in excess.

[1] Archives of Pediatrics, February 1901.

